# Derivation and Validation of the Potential Core Genes in Pancreatic Cancer for Tumor-Stroma Crosstalk

**DOI:** 10.1155/2018/4283673

**Published:** 2018-11-05

**Authors:** Ran Xue, Lin Hua, Wenbin Xu, Yan Gao, Yanhua Pang, Jianyu Hao

**Affiliations:** ^1^Department of Gastroenterology, Beijing Chao-Yang Hospital, Capital Medical University, Beijing 100020, China; ^2^College of Biomedical Engineering, Capital Medical University, Beijing 100069, China

## Abstract

**Background:**

Pancreatic cancer is a fatal malignancy with a poor prognosis. The interactions between tumor cells and stromal cells contribute to cancer progression. Pancreatic stellate cells (PSCs) play a key role in tumor-stroma crosstalk of pancreatic cancer. The in-depth exploration for tumor-stroma crosstalk is helpful to develop novel therapeutic strategies. Our aim was to identify the potential core genes and pathways in tumor-stroma crosstalk.

**Methods:**

3 microarray datasets were from Gene Expression Omnibus (GEO). Differentially expressed genes (DEGs) were screened through bioinformatics analysis. Gene Ontology (GO) enrichment, Kyoto Encyclopedia of Genes and Genomes (KEGG) enrichment, and protein-protein interaction (PPI) network were used to obtain the biological roles of DEGs. The top 15 DEGs were explored by principal component analysis. We validated the top 15 DEGs expression in the tumor-stroma crosstalk model in which PSCs were treated with the mixture of Aspc-1 and Panc-1 supernatant.

**Results:**

A total of 221 genes were filtered as DEGs for tumor-stroma crosstalk. The results of principal component analysis for the top 15 DEGs can distinguish three groups. According to the KEGG enrichment, there were 8, 7, and 7 DEGs enriched in cancer related pathway, PI3K-Akt signaling pathway, and microRNAs, respectively. In the tumor-stroma crosstalk model, significant differences can be validated in the AKAP12, CLDN1, CP, FKBP1A, LAMB3, LSM4, MTMR3, PRKARIA, YWHAZ, and JUND expressions.

**Conclusions:**

These results identified the potential core genes and pathways in pancreatic cancer for tumor-stroma crosstalk, which could provide potential targets for the treatment of pancreatic cancer.

## 1. Background

Accompanied with nearly 100% of 5-year mortality rate, pancreatic cancer is one of the most quickly fatal cancers around the world [1]. Although in recent year we have some amazing improvements in the surgery, radiation therapy, and chemotherapy, pancreatic cancer still has a desperate prognosis [2]. It is one of the main causes for clinical treatment difficulties that pathogenesis and development of pancreatic cancer are not fully understood [3]. Thus, an in-depth exploration into the molecular mechanism of pancreatic cancer biology is urgently needed to develop effective therapeutic approaches.

Cancer is not only actuated by the accumulation of variety of somatic aberrations, but also accelerated by the interaction between cancer cells and the ambient microenvironment [4]. The tumor microenvironment consists of a variety of cell types, such as immune cells, pericytes, fibroblasts, bone-marrow-derived cells, and vascular endothelial cells, embedded in the extracellular matrix (ECM). In recent years, the opinion that stromal cells contribute a great effort to tumor initiation and progression was extensively accepted [5]. Cancer-associated fibroblasts (CAFs) can induce the tumorigenesis through ECM remodeling, angiogenesis, and the secretion of soluble factors.

Remarkable desmoplasia is the pathological feature of pancreatic cancer and leads to its malignant potential. Desmoplasia includes an excessive amount of ECM, which inhibits drug delivery to tumor cells, resulting in chemoresistance [6]. Now, several therapeutic agents have been developed to decrease excessive ECM, such as ECM protein with inhibitor of hyaluronic acid (HA), pegylated recombinant human hyaluronidase (PEGPH20), a novel agent that degrades HA to enhance the delivery of cytotoxic agents, which has demonstrated promising preclinical results and early clinical evidence of efficacy in the first-line treatment of metastatic PDCA with acceptable tolerability. However, for existing therapeutic agents, the potential to augment therapies remains rational next steps and more results of upcoming clinical trials will provide critical guidance [7, 8].

As a main member of CAFs, pancreatic stellate cells (PSCs) are the main sources of ECM production in desmoplasia of pancreatic cancer. In the normal pancreas, PSCs are located between pancreatic lobules and acinar [7]. Under pathological conditions, resident PSCs are activated and secrete excessive amounts of ECM proteins, leading to desmoplasia. There are profound interactions between PSCs and pancreatic cancer cells [8]. Pancreatic cancer cells can stimulate PSCs to produce excessive ECM proteins and cytokines [9]. On the other hand, PSCs also can enhance pancreatic cancer cell proliferation, motility, invasion, and chemoresistance [10]. Based on the evidences above, it is suggested that the tumor-stroma crosstalk of pancreatic cancer is a complicated process. Despite the progress that has been done, the molecular mechanisms of tumor-stroma crosstalk remain unclear. In this study, we used bioinformatics methods to analyze the mRNA expression data during coexposure of pancreatic tumor and PSCs, to identify potential core genes and pathways in tumor-stroma crosstalk, aiming to provide valuable information for further pathogenesis mechanism elucidation and supply the groundwork for therapeutic target identification for pancreatic cancer.

## 2. Materials and Methods

### 2.1. Affymetrix Microarray Data

The source of our data, GSE49583, GSE49584, and GSE49586 transcriptional profile were provided by Giese NA et al. They were in the GEO database (http://www.ncbi.nlm.nih.gov/geo/) from the National Center for Biotechnology Information (NCBI).

### 2.2. Identification of Differentially Expressed Genes (DEGs)

The raw data of the mRNA expression profiles were downloaded and analyzed by R language software. Background correction, probe summarization, and quartile data normalization were applied to the original data. The limma method [11] in Bioconductor (http://www.bioconductor.org/) was utilized to identify DEGs; the significance of DEGs was calculated by t-test and was represented by P value. To reduce the risk of false positives, the Benjamini-Hochberg False Discovery Rate (FDR) method was used for adjusted P values for multiple testing. The corrected P value was represented by FDR. The |log⁡2  FC| > 1.5 and FDR < 0.01 were used as the cut-off criteria. We were interested in the intersection of these sets.

### 2.3. Hierarchical Clustering Analysis of Expression Profiling in PDAC of Tumor-Stroma Crosstalk

To reveal samples in which the most similar groups were neighbors, a two-way hierarchical clustering analysis [12] was applied to genes using the “heatmap” package in the R language. The results were displayed using a heat map.

### 2.4. Gene Ontology Analysis and KEGG Enrichment Pathways

Gene Ontology (GO) [13], Database for Annotation, Visualization and Integrated Discovery (DAVID, http://david.abcc.ncifcrf.gov/) [14], and Kyoto Encyclopedia of Genes and Genomes (KEGG) [15] were used to perform the enrichment analysis including cellular component (CC), molecular function (MF), biological process (BP), and signaling pathway.

The GO-BP terms, GO-CC terms, and GO-MF terms were filtrated by the standard of P value smaller than 0.01 and the significant enriched KEGG pathways were discerned with a P value smaller than 0.05.

### 2.5. Functional Enrichment Analysis of Transcription Factors (TFs)

FunRich (Functional Enrichment analysis tool) from ExoCarta (http://www.exocarta.org/) was used to perform this analysis. As for TFs, the DEGs were chosen and annotated to judge the related TFs. And the cut-off standard was P<0.01.

### 2.6. Principal Component Analysis of the Top 15 DEGs

To analyze the tumor-stroma crosstalk in pancreatic cancer, 15 DEGs with the smallest significant P value were chosen to implement the principal component analysis through R language package pca2d. Principal component analysis is up to determine the major variants in a multidimensional data series [16].

### 2.7. PPI Network Construction and Identification

The context involving molecular mechanism of cellular processing is provided by the functional interactions between proteins. In the current study, DEGs protein-protein interaction (PPI) network was constructed by the Search Tool from the Retrieval of Interacting Genes (STRING, http://string.embl.de/) database and then was visualized through Cytoscape [17].

### 2.8. Isolation and Identification of PSCs and the Construction of Tumor-Stroma Crosstalk Model

Healthy male C57BL/6 mice weighing 13 to 25 g and aged 3 to 11 weeks were used in our tests. Normal C57BL/6 mouse PSCs were primary isolated and cultured using a published method [9]. In detail, pancreatic tissues in C57BL/6 mice were washed by fetal bovine serum, minced into 0.4-1.0 mm3, and then cultured in sterile culture flasks. When they reached 70%-85% confluence after 4-5 days, primary PSCs were collected and then passed on. Primary mouse PSCs were used in the second to sixth passage of this study.

The changes in intracellular lipid droplets were detected using oil red O staining (Sigma-Aldrich); *α*-SMA and desmin expression were tested by immunocytofluorescent staining. Intracellular lipid droplets, *α*-SMA, and desmin expression were used to identify primary PSCs. The list of primary antibodies was shown in Supplementary Table S1 online.

Isolated primary C57BL/6 mice PSCs were treated by DMEM with 15%FBS containing the mixture of Aspc-1 and Panc-1 supernatant for 72 hours as the tumor-stroma crosstalk model.

### 2.9. Real-Time RT-QPCR

According to the manufacturer's instructions, quantitative real-time RT-PCR assessment was conducted by Prime Script RT-PCR kits (RR820A and RR047A, Takara). The expression of HPRT was used as an internal control. The expression of each gene was normalized to that of HPRT. Fold-induction was calculated via the 2−ΔΔCT method [18].

### 2.10. Western Blotting Analysis

Western blotting analysis was performed as we described above [9]. Protein concentrations were measured by the bicinchoninic acid (BCA) Protein Assay Kit (P0010, Beyotime Biotechnology, China). Samples of 15 *μ*g total protein were used for western blotting. The list of primary antibodies was shown in Supplementary Table S1 online.

### 2.11. The Top 15 DEGs Traits in Human Pancreatic Cancer Tissue

The top 15 DEGs protein expressions in pancreatic cancer tissues were determined from the human protein atlas (www.proteinatlas.org).

### 2.12. Statistical Analysis

Data are shown as means ± SD. All statistical analyses were performed via SPSS 16.0 for Windows (SPSS Inc., IL, USA). A two-tailed Student's t-test or one-way ANOVA was used to contrast intergroup variance. The P value of < 0.05 was considered statistically important. 

## 3. Results

### 3.1. Identification of DEGs and Hierarchical Clustering Analysis of Expression Profiling in Tumor-Stroma Crosstalk

Total 28 samples are the expression data from primary human PSCs treated with 8 tumor cell lines, MiaPaCa2 cell treated with human naïve PSCs and MiaPaCa2 treated with human prestimulated PSCs, respectively. The samples (named from GSM1202262 to GSM1202279 and from GSM1202282 to GSM1202291) were found gathered in pancreatic cancer in tumor-stroma crosstalk clusters (Supplementary Figure S1 online). 28 samples were grouped into three clusters by hierarchical clustering. There were 221 DEGs in the intersection. The top 15 DEGs with the minimum significant P value genes were listed in Table 1.

### 3.2. GO Analysis of Identified DEGs

To gain insight into the functional characteristics of identified 221 DEGs, we conducted GO enrichment analyses. As shown in Table 2, positive regulation of transcription from an RNA polymerase II promoter (GO: 0045944, P=2.34E-05), antigen processing and presentation of peptide antigen via MHC class I (GO: 0002474, P=3.00E-04), and mitophagy (GO: 0000422, P=5.49E-04) were the most significant enrichments of GO-BP. Under GO-MF, the genes were enriched in transcription factor activity, sequence-specific DNA binding (GO: 0003700, P=1.06E-04), transcription factor binding (GO: 0008134, P=2.65E-04), and protein binding (GO: 0005515, P=3.06E-04). Moreover, GO-CC analysis revealed genes significantly enriched in focal adhesion (GO: 0005925, P=9.71E-04), endoplasmic reticulum (GO: 0005783, P=1.30E-03), and Golgi apparatus (GO: 0005794, p=2.08E-03). These significantly enriched terms could help us to further understand the role of DEGs in pancreatic cancer occurrence and progress.

### 3.3. Functional Enrichment Analysis of TFS

The TFs of identified 221 DEGs were significantly enriched in HSF1, PLAU, ATF1, EGF1, and ZFG161 (all p<0.001). SP1 is 15.1% in all transcription factor enrichment analyses. As a zinc finger transcription factor, SP1 binds to GC-rich motifs of many promoters. All TFs for coexpressed DEGs were shown in Supplementary Figure S2 online.

### 3.4. KEGG Pathway Enrichment of Identified DEGs

According to the KEGG pathway-related database, there were 8, 7, 7, 6, 6, 5, 5, 4, and 4 DEGs enriched in pathways in cancer, PI3K-Akt signaling pathway, microRNAs in cancer, lysosome, IL-17 signaling pathway, pancreatic cancer, MAPK signaling pathway, P53 signaling pathway, and TNF signaling pathway, respectively. Meanwhile, the results of pathway enrichment analysis via DAIVD were shown in Table 3.

### 3.5. Principal Component Analysis of the Top 15 DEGs

The results of principal component analysis for the top 15 DEGs can distinguish the pancreatic cancer cells with prestimulated PSCs, pancreatic cancer cells with naïve PSCs and prestimulated PSCs directly (Supplementary Figure S3 online).

Comp.1=-0.195LSM4-0.272MTMR3-0.277KLRC3-0.272CLDN1-0.274SAR1B-0.276YMHAZ-0.277ZBTB33-0.273STYX-0.257JUND-0.277FKBP1A-0.268S100Z-0.277CP-0.251LAMB3-0.277PRKAR1A

Comp.2=0.504LSM4-0.140MTMR3+0.115CLDN1-0.698AKAP12+0.136STYX-0.265 JUND-0.183 S100Z-0.302 LAMB3

The first constituted principal component explained 85.97% of the variance of 14 variables, the second principal component explained 12.94% of the variance of 1 variable, and the cumulative variance explained is 98.91%. It is indicated that the top 15 DEGs have a great effect in tumor-stroma crosstalk.

### 3.6. PPI Network Construction

**Figure 1:**
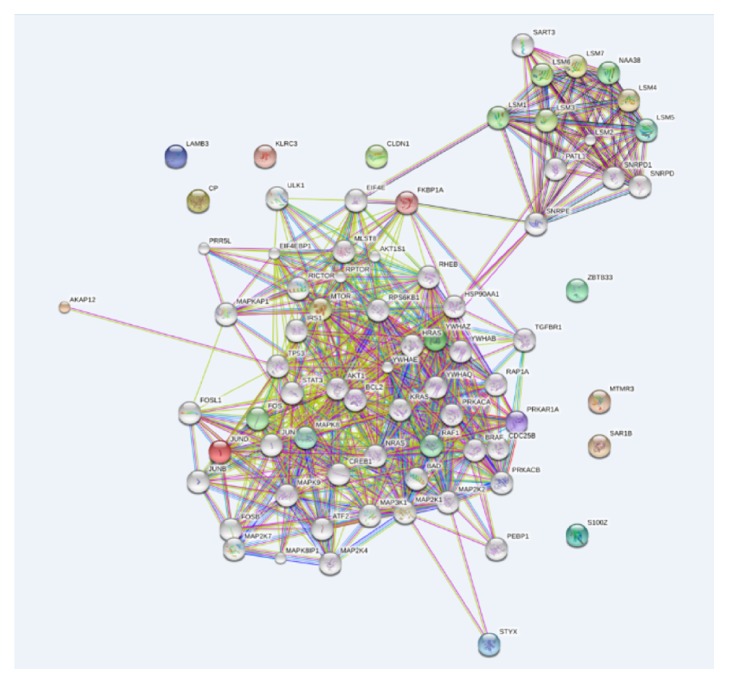
PPI network of the top 15 DEGs. The lines represent the protein-protein interaction relationships corresponding to the genes.

PPI network analysis can identify the key hub members among a cluster of molecules. Based on STRING database, a PPI network of the top 15 DEGs was constructed. The network consisted of 75 nodes and 675 edges. Average node degree was 18. Clustering coefficient was 0.772. As shown in Figure 1, no interaction of CP, LAMB3, CLDN1, ZBTB33, MTMR3, S100Z, and KLRC3 with other proteins was showed, while the rest 8 DEGs constituted 60 PPI pairs. PPI enrichment P value was less than 0.01.

A PPI network of 221 DEGs was also constructed. The network consisted of 274 nodes and 1261 edges. Average node degree was 9.2. Clustering coefficient was 0.736. PPI enrichment P value was 6.76E-12 (Supplementary Figure S4 online).

### 3.7. Characterization of PSCs

**Figure 2:**
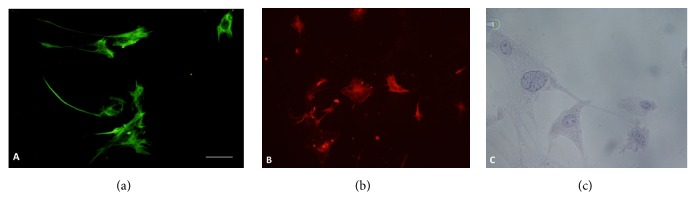
(a): PSCs stain positively for desmin by IF. Original magnification, x200. (b): PSCs stain positively for *α*-SMA by IF. Original magnification, x200. (c): PSCs contain lipid droplets by oil red O staining. Original magnification, x200.

For 5 days, the primary PSCs cells crawled out. The cells contained lipid droplets by oil red O staining and stained positively for desmin but negatively for *α*-SMA by IF. When passed on, PSCs were auto-activated, becoming myofibroblast-like cells. Cells were positive for *α*-SMA by IF (Figure 2).

### 3.8. Validation of the Top 15 DEGs Expressions in Tumor-Stroma Crosstalk

**Figure 3:**
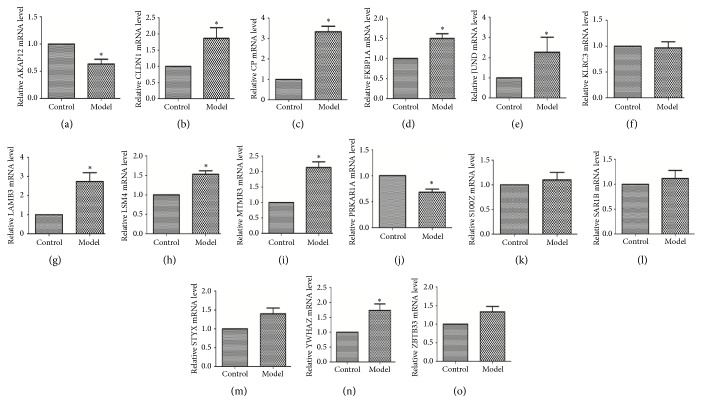
Real-time RT-PCR analysis for 15 top DEGs (*∗*P<0.05, N=3).

**Figure 4:**
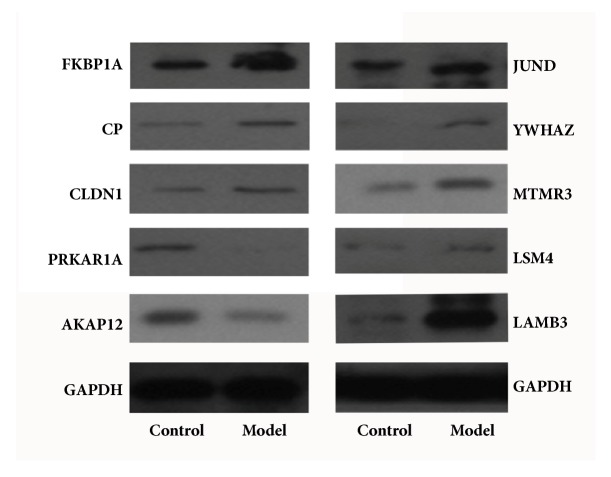
Western blotting analysis for the significant gene of the 15 DEG.

After PSCs were treated with the mixture of Aspc-1 and Panc-1 supernatant for 72 hours, the mRNA of PSCs were extracted to validate the top 15 DEGs expressions. The mRNA levels of the top 15 DEGs were shown in Figure 3. CLDN1, CP, FKBP1A, LAMB3, LSM4, MTMR3, YWHAZ, and JUND mRNA levels were much higher in the tumor-stroma crosstalk. Compared with the control group, PRKARIA and AKAP12 were lower in the tumor-stroma crosstalk. There were no significant differences in SAR1B, ZBTB33, STYX, KLRC3, and S100Z mRNA levels. The significant genes were also identified by western blotting analysis. Our result showed that mRNA and protein data were consistent (Figure 4).

### 3.9. The Characteristics of the Top 15 DEGs in Human Pancreatic Cancer Specimens

**Figure 5:**
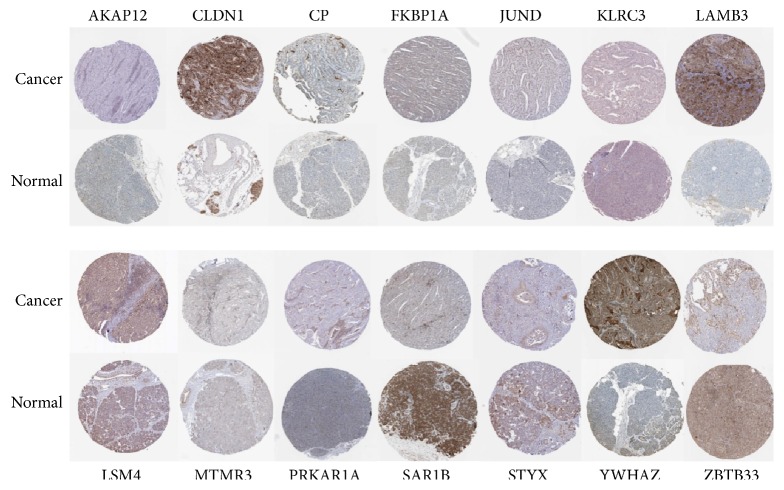
The expression of the top 15 DEGs in human pancreatic cancer specimens. Except for S100Z, the expressions of the other top 14 DEGs in normal pancreas tissue and pancreatic cancer specimens were shown. Images were taken from the Human Protein Atlas (http://www.proteinatlas.org) online database. CLDN1, CP, LAMB3, YWHAZ, PRKARIA, MTMR3, and LSM4 protein levels in pancreatic cancer tissues were higher than that in normal pancreas tissues. Images were available from AKAP12, CLDN1, CP, FKBP1A, JUND, KLRC3, ZBTB33, STYX, SAR1B, LAMB3, YWHAZ, PRKARIA, MTMR3, and LSM4.

To determine the tissue traits of the top 15 DEGs expressions in pancreatic cancer, we analyzed the top 15 proteins expression in clinical specimens from the human protein atlas (www.proteinatlas.org). Except for S100Z, the other 14 DEGs expression profiling in pancreatic cancer specimens was shown in Figure 5. No data of S100Z were reported in human atlas.

## 4. Discussion

Despite advances in medical and surgical therapy, pancreatic cancer still has a poor prognosis. An in-depth exploration into the malignant essence of pancreatic cancer is urgently needed to improve survival rate. There has been increasingly accumulating evidences that support substantial two-way interactions between the stromal components and cancer cells [10, 19, 20]. As a member of stromal cells, PSC plays a vital role in pancreatic fibrosis correlated with pancreatic cancer. Our study identified the potential core genes and pathways in pancreatic cancer for tumor-stroma crosstalk, which is pivotal to the development of new treatment strategies.

Cancer is a complex structure including malignant cells and multifarious surrounding cells. Remarkable desmoplasia is the pathological feature of pancreatic cancer. PSCs are the main sources of ECM production in desmoplasia of pancreatic cancer. Evidence is emerging that there exists a symbiotic relationship between pancreatic cancer cells and PSCs, which lead to an overall increase in the rate of growth of the tumor [21]. Pancreatic cancer cells and PSCs mutually promote each other's differentiation and proliferation [22].

Lately, microarray technology has been widely applied to reveal the genetic alteration in the development of diseases. So, in this study, we used bioinformatics methods to analyze the mRNA expression data during coexposure of pancreatic tumor and PSCs, which were available on the GEO database. In order to gain insight into the functional characteristics of the identified DEGs, we conducted GO, KEGG pathway enrichment analyses, PPI network analysis, and principal component analysis to analyze key genes and pathways in the tumor-stroma crosstalk.

Then, we validated the top 15 DEGs expression in the tumor-stroma crosstalk model. Apart from SAR1B, ZBTB33, STYX, KLRC3, and S100Z, there were significant differences in the other 10 DEGs levels in the tumor-stroma crosstalk. It is supported that they may play a key role in pancreatic cancer for tumor-stroma crosstalk.

CLDN1 is shown to function as a tumor suppressor or oncogene based on some specific cellular context [23]. In pancreatic cancer, CLDN1 can lead to TNF-alpha-dependent proliferation. CLDN1 is also correlated to EMT of pancreatic cancer [24]. It is implied that CLDN1 immunophenotype is closely relevant to the malignant behavior of pancreatic cancer.

Serum levels of Ceruloplasmin (CP) were increased in different tumors. The Capan-1 cells produce CP which contains sLex. Pancreatic cancer cells can result in the synthesis of increased sLex on CP found in patients with pancreatic cancer [25]. Since CP is involved in angiogenesis and neovascularisation of cancer, CP should be a key gene in tumor-stroma crosstalk of pancreatic cancer.

PRKAR1A is a tumor suppressor in the pancreas and points to the PKA pathway as a possible therapeutic target for these lesions. Loss of this gene leads to nonfunctional neuroendocrine tumors with an acinar component. Since loss of PRKAR1A is sufficient to cause endocrine (as well as exocrine) tumorigenesis in the pancreas, PRKAR1A may play a key role in tumor-stroma crosstalk.

LAMB3 can be found in many different kinds of epithelial tissues and tumor microenvironment. It has been reported that LAMB3 may play a significant role in the prognosis and progression of pancreatic cancer. Over-expression of LAMB3 is correlated to clinicopathologic features and reduced survival in pancreatic adenocarcinoma patients [26].

In conclusion, we selected the DEGs and explored the underlying molecular mechanism in pancreatic cancer for tumor-stroma crosstalk by bioinformatics methods. The top 15 DEGs may play a significant role during pancreatic cancer pathogenesis and development. These results will provide valuable information for further molecular mechanism elucidation of tumor-stroma crosstalk in pancreatic cancer. Nevertheless, further experiments are still required.

## Figures and Tables

**Table 1:** The top 15 DEGs with the minimum significant P value genes.

Gene	Gene description
LSM4	LSM4 homolog, U6 small nuclear RNA and mRNA degradation associated
MTMR3	myotubularin related protein 3
KLRC3	killer cell lectin like receptor C3
CLDN1	claudin 1
AKAP12	A-kinase anchoring protein 12
SAR1B	secretion associated Ras related GTPase 1B
YWHAZ	tyrosine 3-monooxygenase/tryptophan 5-monooxygenase activation protein zeta
ZBTB33	zinc finger and BTB domain containing 33
STYX	serine/threonine/tyrosine interacting protein
JUND	JunD proto-oncogene, AP-1 transcription factor subunit
LOC101929368///FKBP1A	uncharacterized LOC101929368///FK506 binding protein 1A
S100Z	S100 calcium binding protein Z
CP	ceruloplasmin (ferroxidase)
LAMB3	laminin subunit beta 3
PRKAR1A	protein kinase cAMP-dependent type I regulatory subunit alpha

**Table 2:** Gene ontology functional enrichment analysis for 221 DEGs.

**Term**	**GO ID**	**Count**	**Fold Enrichment**	**P value**
**GO biological process **				
positive regulation of transcription from RNA polymerase II promoter	GO:0045944	27	2.511767052	2.34E-05
antigen processing and presentation of peptide antigen via MHC class I	GO:0002474	5	15.21014493	3.00E-04
mitophagy	GO:0000422	5	13.03726708	5.49E-04
negative regulation of transcription from RNA polymerase II promoter	GO:0000122	19	2.408272947	9.04E-04
negative regulation of transcription, DNA-templated	GO:0045892	15	2.743312712	1.16E-03
transcription from RNA polymerase II promoter	GO:0006366	15	2.668446479	1.51E-03
response to cAMP	GO:0051591	5	9.919659735	1.56E-03
cellular response to calcium ion	GO:0071277	5	8.947144075	2.29E-03
response to cytokine	GO:0034097	5	8.775083612	2.46E-03
apoptotic process	GO:0006915	15	2.414308719	3.75E-03
antigen processing and presentation of exogenous peptide antigen via MHC class I, TAP-independent	GO:0002480	3	30.42028986	4.04E-03
positive regulation of protein insertion into mitochondrial membrane involved in apoptotic signaling pathway	GO:1900740	4	12.16811594	4.16E-03
positive regulation of transcription, DNA-templated	GO:0045893	14	2.480878008	4.30E-03
cell cycle arrest	GO:0007050	7	4.530681468	4.50E-03
positive regulation of apoptotic process	GO:0043065	10	3.042028986	5.72E-03
regulation of cell death	GO:0010941	3	24.88932806	6.09E-03
interferon-gamma-mediated signaling pathway	GO:0060333	5	6.4268218	7.52E-03
positive regulation of cell differentiation	GO:0045597	4	9.866039953	7.54E-03
cellular response to DNA damage stimulus	GO:0006974	8	3.510033445	7.87E-03
macroautophagy	GO:0016236	5	6.004004577	9.52E-03
$\underline{GO}$ **cellular component**				
focal adhesion	GO:0005925	13	3.123263111	9.71E-04
endoplasmic reticulum	GO:0005783	20	2.269037303	1.30E-03
Golgi apparatus	GO:0005794	20	2.177013774	2.08E-03
ER to Golgi transport vesicle membrane	GO:0012507	5	9.032513878	2.22E-03
cytosol	GO:0005829	52	1.473539519	2.81E-03
integral component of lumenal side of endoplasmic reticulum membrane	GO:0071556	4	12.95698542	3.49E-03
PML body	GO:0016605	6	5.751314959	3.88E-03
melanosome	GO:0042470	6	5.580483822	4.41E-03
membrane	GO:0016020	37	1.579868791	5.09E-03
MHC class I protein complex	GO:0042612	3	25.61949391	5.76E-03
$\underline{GO}$ **molecular function**				
transcription factor activity, sequence-specific DNA binding	GO:0003700	25	2.399737296	1.06E-04
transcription factor binding	GO:0008134	12	3.897714154	2.65E-04
protein binding	GO:0005515	119	1.249546078	3.06E-04
enzyme binding	GO:0019899	12	3.324176636	9.98E-04
transcriptional activator activity, RNA polymerase II core promoter proximal region sequence-specific binding	GO:0001077	10	3.908724646	1.07E-03
chaperone binding	GO:0051087	6	6.833029751	1.82E-03
DNA binding	GO:0003677	32	1.763362516	1.94E-03
RNA polymerase II core promoter proximal region sequence-specific DNA binding	GO:0000978	11	2.858323713	5.22E-03
transcription factor activity, RNA polymerase II distal enhancer sequence-specific binding	GO:0003705	5	6.988325882	5.60E-03
transcriptional repressor activity, RNA polymerase II transcription factor binding	GO:0001191	3	23.06147541	7.11E-03
identical protein binding	GO:0042802	17	2.093698702	7.18E-03
RNA polymerase II activating transcription factor binding	GO:0001102	4	9.710094909	7.88E-03
transcription regulatory region DNA binding	GO:0044212	8	3.464634803	8.42E-03
neurexin family protein binding	GO:0042043	3	19.76697892	9.66E-03

**Table 3:** The enriched KEGG pathways for 221 DEGs.

KEGG terms	KEGG ID	Count	$\underline{P\;\;value}$	$\underline{Fold\;\;Enrichment}$	Genes
HTLV-I infection	hsa05166	12	3.36E-04	3.68	ZFP36, FOS, CDKN2A, XIAP, RELA, TGFBR1, TP53, HLA-C, RB1, HLA-B, CANX, HLA-G
Viral carcinogenesis	hsa05203	10	1.03E-03	3.83	YWHAZ, CDKN2A, HIST2H2BE, RELA, TP53, HLA-C, RB1, HLA-B, YWHAE, HLA-G
Osteoclast differentiation	hsa04380	7	5.96E-03	4.20	FOS, SQSTM1, RELA, TGFBR1, JUND, FOSB, JUNB
Pancreatic cancer	hsa05212	5	8.86E-03	6.04	CDKN2A, RELA, TGFBR1, TP53, RB1
Epstein-Barr virus infection	hsa05169	8	9.74E-03	3.31	YWHAZ, RELA, TP53, HLA-C, RB1, HLA-B, YWHAE, HLA-G
Chronic myeloid leukemia	hsa05220	5	1.26E-02	5.45	CDKN2A, RELA, TGFBR1, TP53, RB1
Antigen processing and presentation	hsa04612	5	1.51E-02	5.17	KLRC3, HLA-C, HLA-B, CANX, HLA-G
Type I diabetes mellitus	hsa04940	4	1.55E-02	7.48	CPE, HLA-C, HLA-B, HLA-G
Lysosome	hsa04142	6	1.79E-02	3.89	GNS, TPP1, PSAP, SORT1, SCARB2, ENTPD4
Small cell lung cancer	hsa05222	5	2.19E-02	4.62	LAMB3, XIAP, RELA, TP53, RB1
Hepatitis B	hsa05161	6	3.56E-02	3.25	FOS, YWHAZ, RELA, TGFBR1, TP53, RB1
Hippo signaling pathway	hsa04390	6	4.12E-02	3.12	YWHAZ, TGFBR1, SERPINE1, TEAD2, YWHAE, FBXW11
Chagas disease (American trypanosomiasis)	hsa05142	5	4.17E-02	3.78	GNAL, FOS, RELA, TGFBR1, SERPINE1
TNF signaling pathway	hsa04668	5	4.42E-02	3.70	FOS, CEBPB, CXCL3, RELA, JUNB

## Data Availability

The source of our data, GSE49583, GSE49584, and GSE49586 transcriptional profile were provided by Giese NA et al. They were in the GEO database (http:// www.ncbi.nlm.nih.gov/geo/) from the National Center for Biotechnology Information (NCBI).

## Abbreviations

PSCs: Pancreatic stellate cells
GEO: Gene Expression Omnibus
DEGs: Differentially expressed genes
GO: Gene Ontology
KEGG: Kyoto Encyclopedia of Genes and Genomes
PPI: Protein-protein interaction
ECM: Extracellular matrix
CAFs: Cancer-associated fibroblasts
CC: Cellular component
MF: Molecular function
BP: Biological process
TFs: Transcription factors.

## Supplementary Materials

Supplementary Materials Supplementary Figure S1 online: hierarchical clustering diagram of differences in pancreatic cancer. The horizontal axis represents the sample name, total 28 samples named from GSM1202262 to GSM1202279 and from GSM1202282 to GSM1202291. The right ordinate axis represents the clustering condition of genes; the upper horizontal axis represents the clustering situation of the sample. 28 samples can be grouped into three clusters through hierarchical clustering, that is, the pancreatic cancer with prestimulated PSCs, pancreatic cancer with naïve PSCs, and prestimulated PSCs, respectively. Supplementary Figure S2 online: functional enrichment analysis of transcription factors (TFs). The transcription factors (TFs) enrichment analysis for 221 DEGs was shown. The TFs for coexpressed DEGs were mainly involved in HSF1, PLAU, ATF1, EGF1, and ZFG161. Supplementary Figure S3 online: the results of two-dimensional principal component analysis of the top 15 DEGs. The results of principal component analysis showed that the sample can be divided into three groups by the top 15 DEGs, pancreatic cancer with prestimulated PSCs, pancreatic cancer with naïve PSCs, and prestimulated PSCs, respectively. Supplementary Figure S4 online: PPI Network of 221 DEGs. The lines represent the protein-protein interaction relationships corresponding to the genes. Supplementary Table S1 online: the antibodies and conditions used in this study.
