# Supplementary material for: Derivation and Validation of the Potential Core Genes in Pancreatic Cancer for Tumor-Stroma Crosstalk
Source: Biomed Res Int. 2018 Nov 5;2018:4283673. doi: 10.1155/2018/4283673 (PMC6241336; doi:10.1155/2018/4283673)
Supplement: Supplementary Materials — Supplementary Figure S1 online: hierarchical clustering diagram of differences in pancreatic cancer. The horizontal axis represents the sample name, total 28 samples named from GSM1202262 to GSM1202279 and from GSM1202282 to GSM1202291. The right ordinate axis represents the clustering condition of genes; the upper horizontal axis represents the clustering situation of the sample. 28 samples can be grouped into three clusters through hierarchical clustering, that is, the pancreatic cancer with prestimulated PSCs, pancreatic cancer with naïve PSCs, and prestimulated PSCs, respectively. Supplementary Figure S2 online: functional enrichment analysis of transcription factors (TFs). The transcription factors (TFs) enrichment analysis for 221 DEGs was shown. The TFs for coexpressed DEGs were mainly involved in HSF1, PLAU, ATF1, EGF1, and ZFG161. Supplementary Figure S3 online: the results of two-dimensional principal component analysis of the top 15 DEGs. The results of principal component analysis showed that the sample can be divided into three groups by the top 15 DEGs, pancreatic cancer with prestimulated PSCs, pancreatic cancer with naïve PSCs, and prestimulated PSCs, respectively. Supplementary Figure S4 online: PPI Network of 221 DEGs. The lines represent the protein-protein interaction relationships corresponding to the genes. Supplementary Table S1 online: the antibodies and conditions used in this study. [file 4283673.f1.docx]

Supplementary Figure S1 online

Hierarchical Clustering Diagram of Differences in pancreatic cancer

The horizontal axis represents the sample name, total 28 samples named from GSM1202262 to GSM1202279 and from GSM1202282 to GSM1202291. The right ordinate axis represents the clustering condition of genes; the upper horizontal axis represents the clustering situation of the sample. 28 samples can be grouped into three clusters through hierarchical clustering. That is the pancreatic cancer with pre-stimulated PSCs, pancreatic cancer with naïve PSCs and pre-stimulated PSCs respectively.


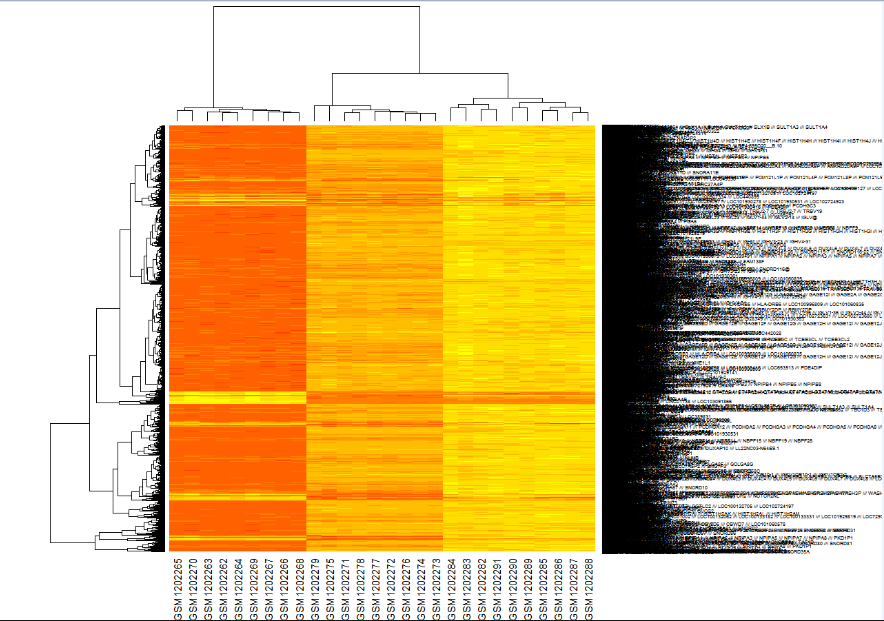


Supplementary Figure S2 online

Functional Enrichment analysis of transcription factors (TFs)

The transcription factors (TFs) enrichment analysis for 221 DEGs. The TFs for co-expressed DEGs were mainly involved in HSF1, PLAU, ATF1, EGF1 and ZFG161.


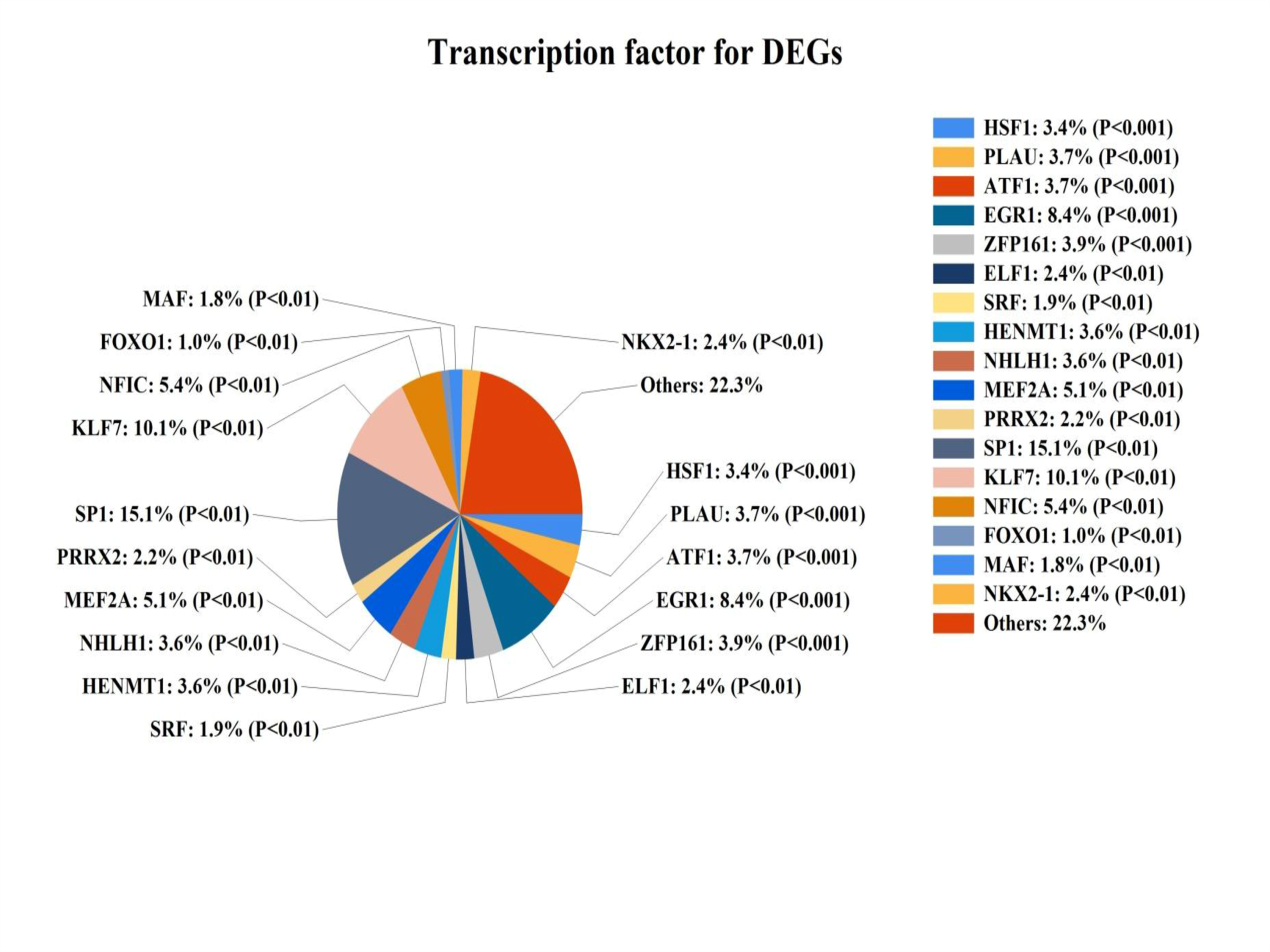


Supplementary Figure S3 online

The results of two - dimensional principal component analysis of the top 15 DEGs

The results of principal component analysis showed that the sample can be divided into three groups by the top 15 DEGs, pancreatic cancer with pre-stimulated PSCs, pancreatic cancer with naïve PSCs and pre-stimulated PSCs, respectively.


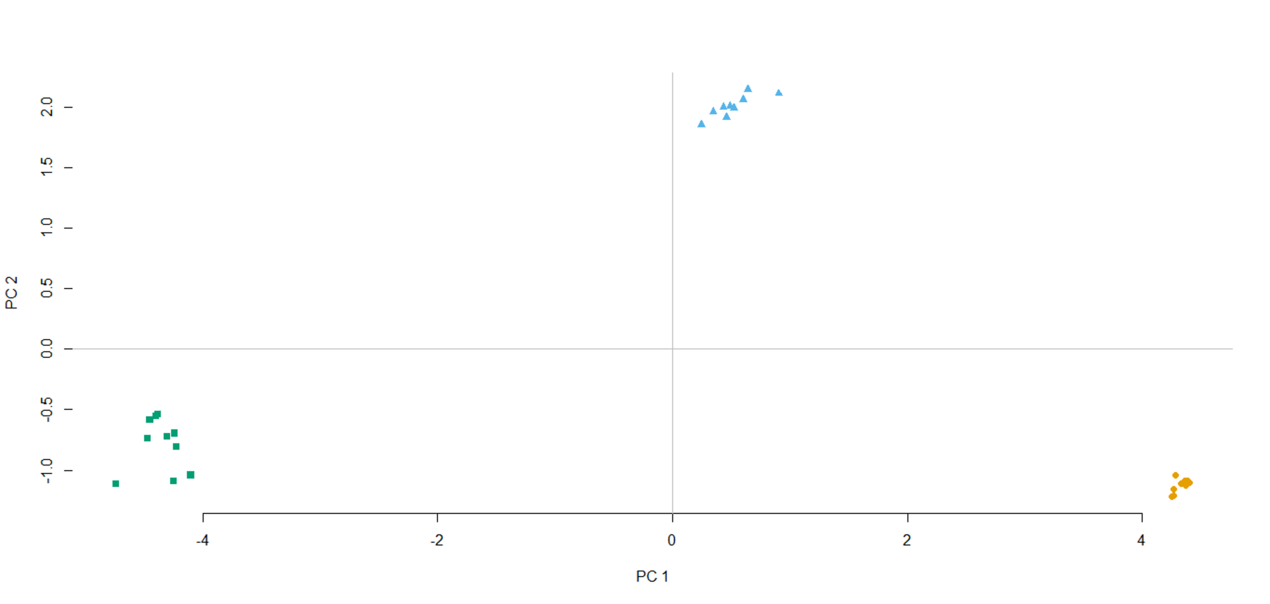


Supplementary Figure S4 online

PPI Network of 221 DEGs.

The lines represent the protein-protein interaction relationships that corresponding to the genes.


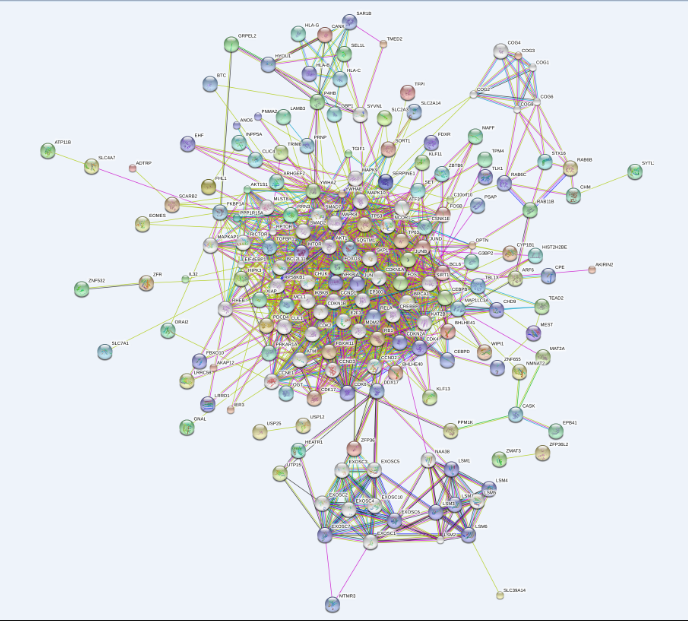


Supplementary Table S1 online

The antibodies and conditions used in this study.

| Antibody | Number | Species | Dilution | Source |
| --- | --- | --- | --- | --- |
| α-SMA | ab5694 | Rabbit polyclonal antibody | 1:500 | Abcam |
| Desmin | ab32362 | Rabbit monoclonal antibody | 1:1000 | Abcam |
| CLDN1 | ab211737 | Rabbit polyclonal antibody | 1:1000 | Abcam |
| CP | ab48614 | Rabbit polyclonal antibody | 1:1000 | Abcam |
| FKBP1A | Ab2918 | Rabbit polyclonal antibody | 1:1000 | Abcam |
| LAMB3 | Ab97765 | Rabbit polyclonal antibody | 1:1000 | Abcam |
| GAPDH | #5174 | Rabbit monoclonal antibody | 1:1000 | Cell Signaling Technology |
| LSM4 | Ab153959 | Rabbit polyclonal antibody | 1:1000 | Abcam |
| MTMR3 | #12443 | Rabbit monoclonal antibody | 1:1000 | Cell Signaling Technology |
| YWHAZ | ab137869 | Rabbit monoclonal antibody | 1:1000 | Abcam |
| JUND | #5000 | Rabbit monoclonal antibody | 1:1000 | Cell Signaling Technology |
| PRKARIA | ab75749 | Rabbit monoclonal antibody | 1:1000 | Abcam |
| AKAP12 | #9272 | Rabbit monoclonal antibody | 1:1000 | Abcam |
